# The first mitochondrial genome of *Balanus trigonus* Darwin, 1854 (Sessilia: Balanidae) and molecular phylogeny within Cirripedia

**DOI:** 10.1080/23802359.2021.1930216

**Published:** 2021-05-23

**Authors:** Shishi Liu, Sheng Mao, Tian Ge, Yuefeng Cai, Nanjing Ji, Xue Kong, Xin Shen

**Affiliations:** a Jiangsu Institute of Marine Resources, Jiangsu Key Laboratory of Marine Biotechnology, Jiangsu Ocean University, Lianyungang, China; b Co-Innovation Center of Jiangsu Marine Bio-industry Technology, Jiangsu Ocean University, Lianyungang, China

**Keywords:** *Balanus trigonus* Darwin, 1854, mitochondrial genome, gene arrangement, phylogeny

## Abstract

The triangle barnacle *Balanus trigonus* Darwin, 1854, a cosmopolitan inhabitant of tropical and warm temperate seas, is a member of robust system for the study of evolutionary processes in the intertidal zone. The first mitochondrial genome of *B. trigonus* is presented. The complete mitochondrial genome of *B. trigonus* is a circular molecule of 15,560 bp, which encodes 13 protein-coding genes (PCGs), 2 rRNA genes, and 22 tRNA genes. In comparison within Sessilia, the arrangement of the mitochondrial genome of *B. trigonus* is more similar to *Megabalanus* spp. than the congener *Balanus balanus*, which share a same inversion of a large gene block (*P*-*nd4L*-*nd4*-*H*-*nd5*-*F*). Phylogenetic analysis based on mitochondrial PCGs reveals that *B. trigonus* clusters with *Acasta Sulcata* (BP = 100), then grouped with *Megabalanus volcano* and *Megabalanus ajax* with high support (BP = 90). In further, more data and research are needed to reveal the phylogeny within Cirripedia.

The triangle barnacle *Balanus trigonus* Darwin (1854), a cosmopolitan inhabitant of tropical and warm temperate seas, is widely distributed in the southeast coastal areas of China. As a common indicator of the coastal waters, *B. trigonus* occupies an important place in fouling acorn barnacle community in high-salt waters, it is a robust system for the study of evolutionary processes in the intertidal zone because of its large percentage of the total biomass of fouling organisms (Nunez et al. 2018).

Specimens of *B. trigonus* were collected from Zhoushan Islands in China (30.19°N, 122.70°E), which were deposited in Marine Museum of Jiangsu Ocean University (https://www.jou.edu.cn/, voucher number: Batr-001). The muscle tissue isolated from the fresh specimen was immediately preserved in 95% ethanol. Total DNA was extracted from the muscle tissue with the QIAamp Tissue Kit (Qiagen, Germany) following the manufacturer’s recommendations. The mitochondrial genome of *B. trigonus* was sequenced and annotated according to previous study (Chen et al. 2019).

The mitochondrial genome of *B. trigonus*, with a total length of 15,560 bp, encodes 13 protein-coding genes (PCGs), 2 rRNA genes, and 22 tRNA genes (GenBank Accession no.: MW646099) (Supplementary Table S1). The contents of A, G, T, and C are 33.5%, 12.5%, 38.7%, and 15.3%, respectively (Supplementary Table S2). The length of PCGs is 11,074 bp (70.8%), which was consistent with other available mitochondrial genomes of Balanidae. The total length of non-coding regions is 939 bp, with the longest one as the putative control region (398 bp), which is located between *12S rRNA* and *trnI*. 12 PCGs in *B. trigonus* start with ATD (ATG, ATT or ATA), while *nd4L* starts with GTG. Remarkably, *cox3*, *nd3*, *nd4* and *nd5* ended by T–– as the stop codon and the remaining PCGs have the complete stop codon TAA or TAG. The contents of A + T of *12S rRNA* and *16S rRNA* are 69.8% and 75.8%, respectively. In comparison with Sessilia, the arrangement of the mitochondrial genome of *B. trigonus* is more similar to *Megabalanus* spp. (*Megabalanus ajax*, *Megabalanus volcano*, *Megabalanus tintinnabulum*) than the congener *Balanus balanus*, which share a same inversion of a large gene block (*P*-*nd4L*-*nd4*-*H*-*nd5*-*F*) except a translocation between *trnQ* and *trnC* (Tsang et al. 2017; Shen, Chu, et al. 2016; Shen, Tsoi, et al. 2016; Feng et al. 2019).

To clarify the phylogenetic relationships within Cirripedia, a phylogenetic tree was constructed based on the PCGs from complete mitochondrial genomes with 30 Cirripedia species (all available Sessilia species in NCBI databases and two Pedunculata species as outgroups) used by PhyloSuite software (Shen et al. 2017; Zhang et al. 2020). As shown in Figure 1, B*. trigonus* clusters with *Acasta sulcata* (BP = 100), then groups with *Megabalanus volcano* and *Megabalanus ajax* with high support (BP = 90), which is also supported by the similar arrangement pattern of the mitochondrial genome in the three species. In addition, the phylogenetic tree also shows that the Balanidae, Pyrgomatidae and Archaeobalanidae cluster together as previous studies revealed (Cai et al. 2018; Tian et al. 2020; Mao et al. 2020; Song et al. 2020).

**Figure 1.**
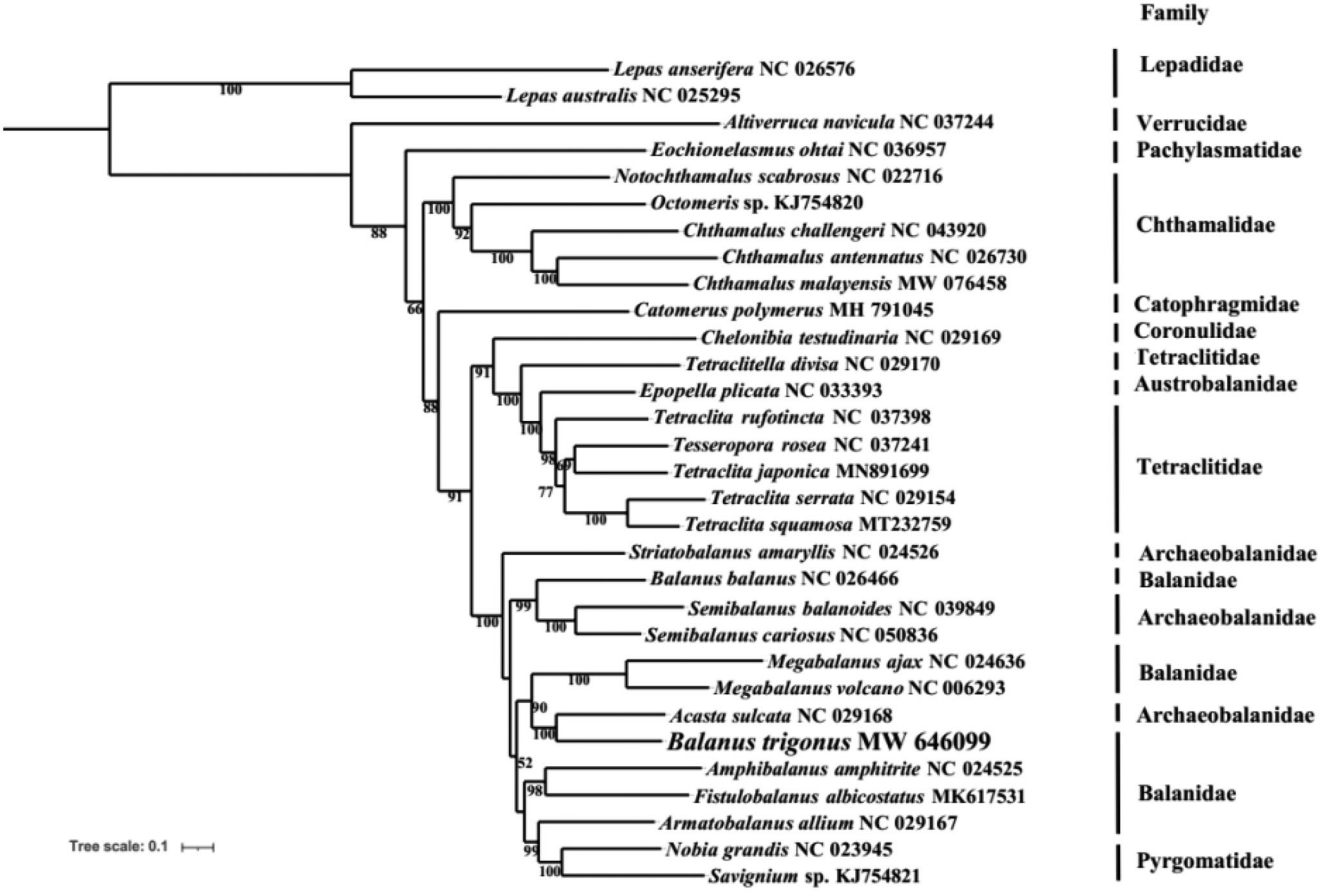
The Maximum-likelihood phylogenetic tree based on 13 PCGs nucleotide sequences of *Balanus trigonus* Darwin, 1854 and other mitochondrial genomes from Cirripedia.

In this study, we report the first mitochondrial genome of *B. trigonus*, which have enriched resources of species within Cirripedia, that provides data support for the phylogeny of Cirripedia.

## Data Availability

The genome sequence data that support the findings of this study are openly available in GenBank of NCBI at (https://www.ncbi.nlm.nih.gov/) under the accession no. MW646099.
